# Editorial: Innovative integration of stress physiology and biotechnological tools for mitigating metal stress in plants

**DOI:** 10.3389/fpls.2026.1854255

**Published:** 2026-05-06

**Authors:** Mohd Irfan Naikoo, Manzoor R. Khan, Stefan John Siebert, Fauzia Naushin

**Affiliations:** 1 Applied Research Centre for Environment and Marine Studies, King Fahd University of Petroleum & Minerals, Dhahran, Saudi Arabia; 2 Department of Botany, University of Kashmir, Baramulla, India; 3 Unit of Environmental Sciences and Management, North-West University, Potchefstroom, South Africa; 4 Department of Botany, Women College, Aligarh Muslim University, Aligarh, India

**Keywords:** metal stress management, metal toxicity, phytoremediation, plant adaptation, resilience mechanisms, soil contamination

Heavy metal contamination of soils remains a persistent, non-biodegradable constraint on plant productivity, ecosystem stability, and food safety, particularly in regions shaped by mining, industrialization and intensive agriculture (Naikoo et al., 2026). Addressing this challenge requires an integrative understanding of plant stress physiology combined with innovative biotechnological strategies. Despite decades of research, mitigation approaches have largely remained fragmented, focusing on isolated physiological, microbial, or geochemical processes. This causes limited translation from controlled experiments to field-scale solutions. The contributions assembled in this Research Topic collectively signal a critical shift toward multi-scale frameworks that link plant stress physiology, rhizosphere ecology, and soil system dynamics.

**Figure 1:**
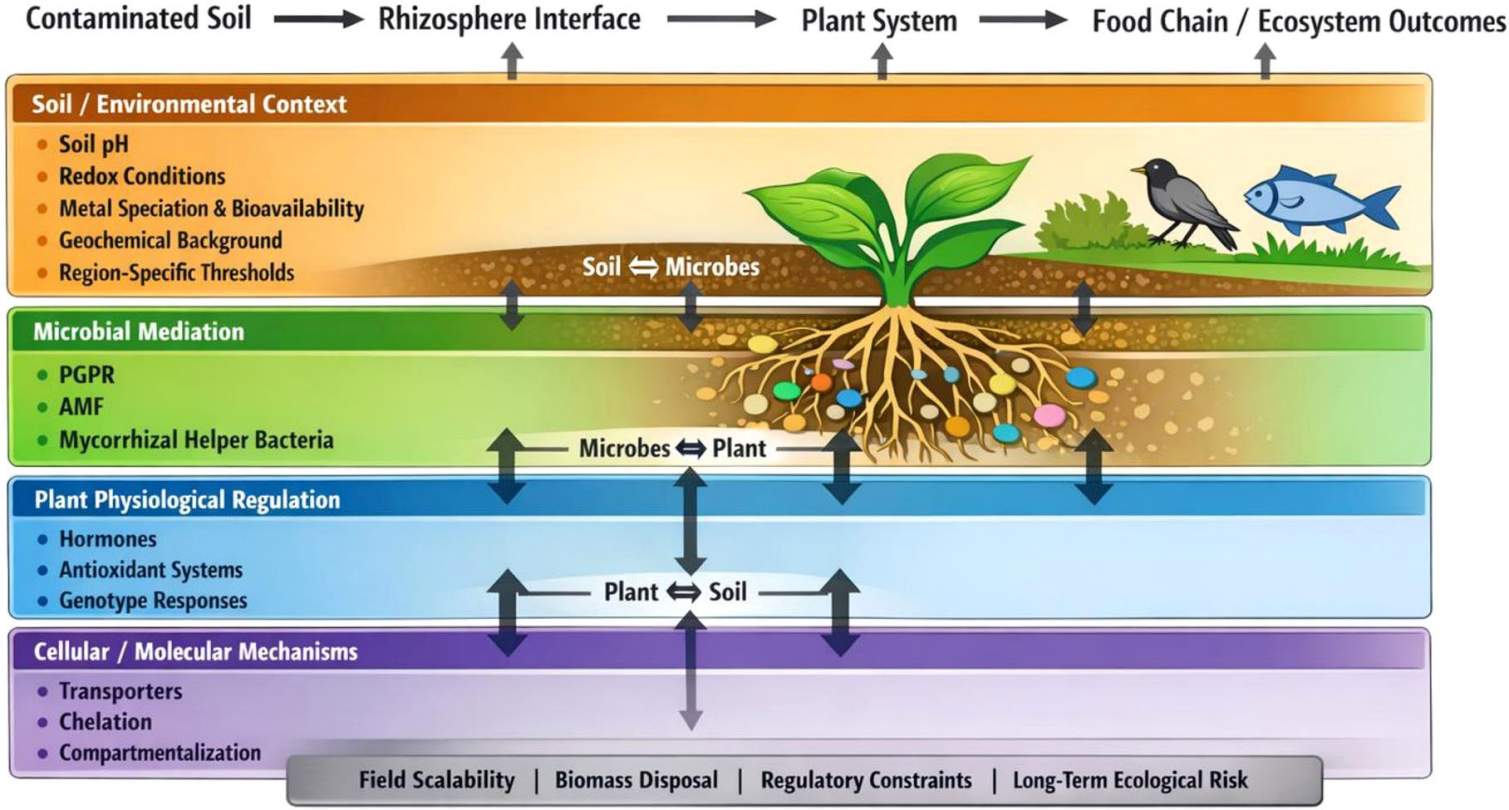
Conceptual framework illustrating the integration of soil properties, microbial processes, plant physiological responses, and molecular mechanisms in mitigating metal stress. The diagram emphasizes cross-scale interactions and feedbacks that govern metal bioavailability, uptake, detoxification, and ecological outcomes, while highlighting key constraints limiting field implementation.

By explicitly connecting molecular mechanisms of metal transport and detoxification with microbial mediation, hormonal regulation, and context-dependent soil processes, these studies advance a systems-level perspective on metal stress mitigation. Rather than treating plant, microbial, and soil processes in isolation, these studies converge on a systems-oriented perspective that links molecular regulation, rhizosphere interactions, and environmental context. This integrative approach links molecular regulation, rhizosphere interactions, and environmental context, providing a basis for translating mechanistic insights into scalable, field-relevant solutions. This perspective is particularly relevant in metal-rich regions of the Global South, where extensive mining activities coincide with highly diverse floras on metalliferous soils. A conceptual synthesis of these cross-scale interactions is presented in **Figure 1**.

Hyperaccumulator plants are one of the main topics covered in this Research Topic. These species possess specialized molecular and physiological traits that enable them to bioaccumulate and sequester large amounts of heavy metals in aerial tissues without exhibiting toxicity symptoms (Bhat et al.). Compared with non-hyperaccumulators, they exhibit enhanced uptake, translocation and compartmentalization capacities, whereas most non-hyperaccumulating species restrict metals to root vacuoles. These capabilities are underpinned by constitutive expression of metal transporter and detoxification genes. Key transport proteins include heavy metal include heavy metal ATPases (HMAs), ATP-binding cassette (ABC) transporters, and natural resistance-associated macrophage proteins (NRAMPs). Detoxification is further facilitated by metallothioneins (MTs) and phytochelatin synthases (PCS), which mediate metal chelation. Under metal stress, genes such as HMA3/4, MT1/2, NRAMP3/4, and PCS1 are frequently upregulated, enabling efficient sequestration and detoxification. These insights not only advance our understanding of plant adaptive evolution, but also inform molecular breeding and biotechnological strategies for phytoremediation.

The synergistic role of beneficial microbes further complements phytoremediation strategies. The combined application of plant growth-promoting rhizobacteria (PGPR) and arbuscular mycorrhizal fungi (AMF) can act as effective bioremediation agents (Amir et al.). AMF extend extraradical hyphal networks into the soil, improving aggregation through glomalin production and enhancing nutrient uptake, whereas PGPR promote plant growth via phytohormone production, nitrogen fixation, ACC deaminase activity, and secretion of metal-chelating compounds. Whenever co-inoculated, these microorganisms display complementary mechanisms that immobilize and sequester metals while enhancing plant tolerance. The role of mycorrhizal helper microbes adds an additional layer of complexity by promoting fungal colonization and function. However, the large-scale field validation remains limited. Translating these findings into practical applications will require multi-location experiments across diverse edaphic and climatic conditions.

A key insight emerging across these contributions is that the effectiveness of any single intervention, whether microbial inoculation, hormonal regulation, or genetic enhancement, is contingent on interactions across organizational scales. Microbial consortia influence metal bioavailability and root uptake, which in turn modulates plant physiological responses and gene expression. These feedbacks are further constrained by soil properties and regional geochemical contexts. Explicitly incorporating such cross-scale interactions is therefore essential for developing robust and predictive mitigation strategies.

Plant hormonal regulation represents another important strategy for mitigating metal stress. Samet and Çıkılı demonstrated nitric oxide (NO) exerts genotype-dependent effects in alleviating cobalt stress in lettuce. Application of sodium nitroprusside (SNP), an NO donor, modulated antioxidant enzyme activities and reduced oxidative stress markers including malondialdehyde (MDA) and hydrogen peroxide (H_2_O_2_). Differential responses between curly and Romaine lettuce varieties highlight the importance of genetic background in determining stress resilience. These results support the idea that physiological interventions need to be customized for particular genotypes and environmental conditions.

Similarly, Al-Amri demonstrated that silicon (Si) and prohydrojasmonate (PDJ) act synergistically to enhance cadmium detoxification in rice. Co-application restored nutrient balance, regulated phytohormonal pathways (abscisic acid (ABA), salicylic acid (SA), and jasmonic acid (JA)) and reduced cadmium translocation from roots to shoots. At the molecular level, genes such as OsLCT1 and OsHMA2, along with detoxifying gene (OsPCS1, OsGSTU5, and OsABCC1), showed distinct expression patterns during co-application of Si and PDJ. These findings demonstrate that mineral and hormonal amendments can be strategically combined to increase crop resilience in contaminated soils.

This Research Topic also highlights the importance of region-specific management of contaminated soils. Wei et al., developed a framework integrating plant physiological responses with soil bioavailability to define region-specific safety thresholds for Cd and As in karst systems. Their findings indicate that soil alkalinity and strong geochemical backgrounds can lead to overestimation of contamination risks when using generic national thresholds. By applying species sensitivity distribution models and multiple regression analyses, they derived more accurate thresholds, thereby reducing needless remediation efforts. This approach underscores the need for context-specific rather than standardized assessments.

Collectively, the studies in this Research Topic demonstrate that effective mitigation of metal stress requires coordinated approaches that integrate plant physiology, microbial ecology, and soil system processes. Advances in genomics and transcriptomics further enhance our ability to identify regulatory networks and underpinning stress tolerance. A promising frontier in the fight against heavy metal contamination is the integration of advanced biotechnology techniques with plant stress physiology. Ecofriendly remediation and resilient agriculture systems are being developed by utilizing the natural inherent resilience of hyperaccumulators, harnessing synergetic microbial properties, pathways of hormone modulation, and improving management frameworks of region-specific contamination.

Despite this progress, key challenges remain, including limited field validation, uncertainties surrounding the management of metal-enriched biomass, and regulatory constraints associated with engineered biological systems. Addressing these limitations will require multidisciplinary collaboration and a stronger emphasis on long-term, field-based research. By moving beyond reductionist approaches toward coordinated, multi-scale strategies, this body of work provides a foundation for developing context-specific and scalable solutions for contaminated landscapes, with direct implications for food security, ecosystem restoration, human health and environmental sustainability.
